# Patients’ and clinicians’ perspectives on the clinical utility of the Rheumatoid Arthritis Foot Disease Activity Index

**DOI:** 10.1007/s00296-022-05147-8

**Published:** 2022-05-27

**Authors:** Anika Hoque, Martijn Steultjens, Diane M. Dickson, Gordon J. Hendry

**Affiliations:** School of Health and Life Sciences, Glasgow Caledonian University, Cowcaddens Road, Glasgow, G4 0BA UK

**Keywords:** Rheumatoid arthritis, Foot, Patient-reported outcome, Qualitative research, Rheumatoid Arthritis Foot Disease Activity Index (RADAI-F5), Observational study, Patient perspective, Clinician perspective

## Abstract

Although patient-reported outcome measures (PROMs) are recommended in clinical practice, their application in routine care is limited. The Rheumatoid Arthritis Foot Disease Activity Index (RADAI-F5) is a validated PROM for assessing foot disease in rheumatoid arthritis (RA). To explore patient and clinician opinions and perceptions of the clinical utility of the Rheumatoid Arthritis Foot Disease Activity Index (RADAI-F5), eight RA patients and eight clinicians routinely involved in the management of RA patients participated in one semi-structured remote video-based interview. They provided their perspectives on the barriers and facilitators to clinical implementation of the RADAI-F5. Three global themes were identified; 'Feet are a priority' as the impact of RA on the feet negatively impacted upon patient quality of life. The second theme was 'Need for a clinically feasible foot PROM' as participants recognised the current lack of a clinically feasible tool to determine RA foot disease. The third global theme of ‘Implementation’ was drawn together to form two subordinate themes: ‘Facilitators to RADAI-F5 implementation’ as the tool can promote communication, guide management, help screen foot symptoms, monitor foot disease status and treatments, and promote patient education and; ‘Barriers to RADAI-F5 implementation’ as there were associated practical difficulties, including lack of appointment time, administrative burdens, IT barriers and preference for further RADAI-F5 validation using imaging. The RADAI-F5 has significant potential as a clinical tool to aid foot disease management. However, implementation challenges must be overcome before broad adoption in rheumatology clinics.

**Supplementary Information:**

The online version contains supplementary material available at 10.1007/s00296-022-05147-8.

## Introduction

Rheumatoid arthritis (RA) is a chronic, systemic, inflammatory condition that typically includes the feet and ankles. Foot disease can cause tenderness, swelling, pain, joint damage, loss of function, and gait issues [1] and can significantly affect personal relationships, psychological well-being, ability to work, and social activities [2, 3]. Evidence-based guidelines recommend that patients with RA should be treated to attain clinical remission or low disease activity [4]. This can be achieved by using composite disease activity measures such as the 28-joint Disease Activity Score 28 (DAS-28), which has commonly been utilised to evaluate the disease status of RA patients [5]. However, the DAS-28 involves reduced joint counts, which exclude the foot and ankle joints. This is mainly attributed to practical considerations such as time constraints during routine appointments and the feet being less accessible for clinical examination than the hands. Additionally, some studies indicate that the DAS-28 is similarly representative of global disease activity as the 44-joint DAS [6]. Nevertheless, emerging evidence suggests that more than one-third of RA patients categorised as in remission by DAS28 had clinically verified active foot synovitis [7, 8]. While the DAS-28 is the most thoroughly validated and extensively used measure of disease activity, the Simplified Disease Activity Index (SDAI) and the Clinical Disease Activity Index (CDAI) are also utilised in clinical and research contexts. Again, these disease activity indicators exclude the foot and ankle joints, and Wechalekar et al. [7] reported that around 25–36% of individuals with SDAI and CDAI remission presented with foot synovitis. As such, relying primarily on the current disease evaluation indices that omit the foot and ankle can result in overlooked foot disease activity, increasing the risk of progressive joint damage and suboptimal foot care. The long-term impact of the feet being omitted from routine RA clinical appointments on patients has not been fully established; however, there is some evidence of structural joint damage and disability in patients with clinically active foot synovitis who are classed as in DAS-28 remission (i.e. < 2.6) [9]. To date, clinicians' reasons for including or omitting foot joints from routine examinations have not yet been explored from a qualitative standpoint.

Studies have demonstrated that a variety of patient-reported outcome measures (PROMs) can accurately assess foot health status, guide medical management and facilitate shared decision-making as they are instruments that obtain information about health issues directly from patient reports [7]. Although the use of PROMs in rheumatological clinical care has been recommended by the American College of Rheumatology (ACR), European League Against Rheumatism (EULAR) and Outcome Measure in Rheumatology Clinical Trials (OMERACT) [10–12], their use is far from common due to a lack of clinical feasibility associated with busy rheumatology clinics and burden on patients as a consequence of their length [13]. As such, a new PROM known as the Rheumatoid Arthritis Foot Disease Activity Index (RADAI-F5) was developed and validated to monitor inflammatory foot disease in individuals with RA. The psychometric features of the RADAI-F5 meet the recommended standards specified by the Consensus-Based Standards for the Selection of Health Measurement Instruments, exhibiting high construct validity, reliability, content validity, internal consistency, responsiveness, and interpretability. This novel instrument has the potential to provide an opportunity for a treat-to-target approach that includes the foot [14].

Patient and clinician perspectives on the barriers and facilitators of integrating the RADAI-F5 into routine clinics have not yet been captured, and these are essential to inform future PROM implementation. While Fung et al. [15] and Boyce et al. [16] described the theoretical barriers to integrating PROMs in clinical care, including logistical and technological constraints, there has been limited comprehensive study from patient and clinician viewpoints, particularly those of rheumatologists and allied health professionals (AHPs) regarding PROM implementation. Understanding the advantages and disadvantages of PROM implementation by patients and clinicians is critical for successfully integrating the RADAI-F5 into the care of patients with chronic systemic conditions. Accordingly, the current study sought to elicit patient and clinician perspectives on using the RADAI-F5 tool to aid in assessing and managing foot disease in RA.

## Materials and methods

### Study design

This study's design was based on interpretive phenomenological analysis (IPA), a qualitative methodology used to understand an individual's lived experiences. This approach is interpretive as the research team uses personal accounts to understand each participant's experience and the broader experience associated with the implementation of a new PROM. Demographic data were collected directly from the participants.

### Compliance with ethical standards

All participants provided written consent prior to each interview, and to protect participants' anonymity, each participant has been assigned a pseudonym. This study received ethical approval from the psychology, social work, and allied health sciences Research Ethics Committee at Glasgow Caledonian University (HLS/PSWAHS/20/096) and all investigations were conducted in conformity with the ethical principles of research.

### Participants

Participants were recruited using a convenience and snowball sampling technique. Patient participants, AHPs, and rheumatologists based in the UK were separated into two groups; patients and clinicians. Patient participants had to be at least 18 years old and have physician-diagnosed RA. Clinicians, such as rheumatologists, rheumatology nurses, rheumatology registrars, physiotherapists, podiatrists, and orthotists, were also eligible if they were routinely involved in treating and managing RA patients. Recruitment took place between February and July 2021. The recruitment process included gatekeepers on behalf of the study team sending e-mails to the membership lists of Versus Arthritis Scotland, the National Rheumatoid Arthritis Society (NRAS), and the Scottish Society for Rheumatology. Furthermore, e-mails were sent out by the principal investigator (AH) to known AHPs and rheumatologists. In addition, the study was advertised on social media using research group accounts.

### Data collection

The principal investigator gathered data using a semi-structured interview guide (Supplemental 1.1 and 1.2). The research team developed the interview guide based on a literature review of previously published research on PROMS and foot disease in RA [15–19]. Five RA patients, two physiotherapists, three podiatrists and one rheumatologist who routinely manage patients with RA-related foot disease also provided additional informal input to inform the development of the interview topic guide. Five pilot interviews were conducted, which allowed testing of the interview questions and interviewing style and approach. There were some minor adaptions to the data-collection instrument as a result of the pilot interviews. The patient and clinician guides were developed exclusively. However, both included open-ended question formats with probes designed to explore patients' overall experience with foot disease, the general use of PROMs in clinical practice, and the clinical utility of the RADAI-F5 in clinical practice. Prior to the interview, patient and clinician participants had the opportunity to review the RADAI-F5 questionnaire to facilitate discussions around the tool. Interviews were conducted by the principal investigator (AH) using video-based calls (Microsoft Teams) or telephone calls. Participants consented to an audio recording. The principle investigator was not involved in the patient care of any patient participants. Data gathering was ceased after 16 interviews when there was enough material for analysis, and no new information arose from subsequent interviews, a phenomenon known as data saturation [20]. Data saturation was determined by transcribing after each interview until no new themes emerged and at this point, recruitment was discontinued. Demographic and clinical data, including age, gender, disease duration and years of clinical experience, were also collected. Digital audio recordings from each session were transcribed verbatim, and transcriptions were uploaded into Nvivo Software (version 12, QSR, Melbourne) [21] for analysis.

### Data analysis

To ensure the accuracy of the data, twelve participants read and validated the transcripts within one week of their interview. Data was analysed thematically using principles of IPA and primarily followed the stages indicated by Smith et al. [22]. The principal researcher (AH) first read the transcripts, created codes, and noted discussion points. Each transcript was analysed separately to maintain the individual's perspective and develop themes that emerged from participants' accounts of their experiences and views. The process included the identification of essential themes by an open coding process. The codes were derived from the data's lowest order themes. The codes were then organised into groups and developed into emerging themes. In the next stage, the goal was to reduce the volume of data while maintaining the robustness of the participant's narrative. These emergent themes were brought together in an iterative process that entailed grouping and re-grouping seemingly related emerging themes until an overall understanding was reached. Gradually, categories were consolidated into themes. Four transcripts were also separately analysed by study team members (GJH, DMD and MS). Emerging themes were reviewed with these study team members to identify any new topics of interest, and where there was disagreement, overall emergent themes were resolved and agreed upon through further discussion. The teams' process of theme verification provided varied perspectives and agreement on final themes, which increased the credibility and validity of the study results using a robust approach [23].

## Results

### Participant characteristics

Interviews lasted between 35 and 70 min and were held between 9th March and 9th July 2021. In total, 12 individuals with RA and 11 clinicians were contacted to participate in this study. Of these, reasons for non-participation included: time constraints (*n* = 2)*,* changed their mind (*n* = 1) and being unable to contact further after initial contact (*n* = 4). Eight RA patient participants: 7 females; median [Interquartile range(IQR)] age of 54 [10.5], median [IQR] disease duration of 11 [14] years and eight clinicians; median [IQR] age 44.5 [8], median [IQR] years of clinical experience of 20 [3] participated in video-based calls. Demographic details of RA participant characteristics are presented in Table 1, and clinician characteristics are presented in Table 2.

**Table 1:** RA participant characteristics

Participant ID	Gender	Age (years)	Disease duration (Years)	Current medication
P01	Female	61	27	Biologics
P02	Female	40	15	Biologics
P03	Male	68	9	DMARD, Biologics
P04	Female	50	12	Biologics
P05	Female	58	56	DMARD
P06	Female	48	5	DMARDs
P07	Female	57	10	DMARD, Biologics
P08	Female	51	3	DMARDs

*DMARD* disease-modifying antirheumatic drugs

**Table 2:** Clinician participant characteristics

Participant ID	Gender	Age (years)	Profession	Years of clinical experience
C10	Female	43	Podiatrist	21
C11	Male	45	Rheumatologist	20
C12	Male	44	Podiatrist	20
C13	Female	56	Podiatrist	16
C14	Male	49	Podiatrist	16
C15	Male	44	Physiotherapist	20
C16	Male	54	Rheumatologist	22
C17	Female	39	Podiatrist	21

### Overview of themes

**FIG. 1:**
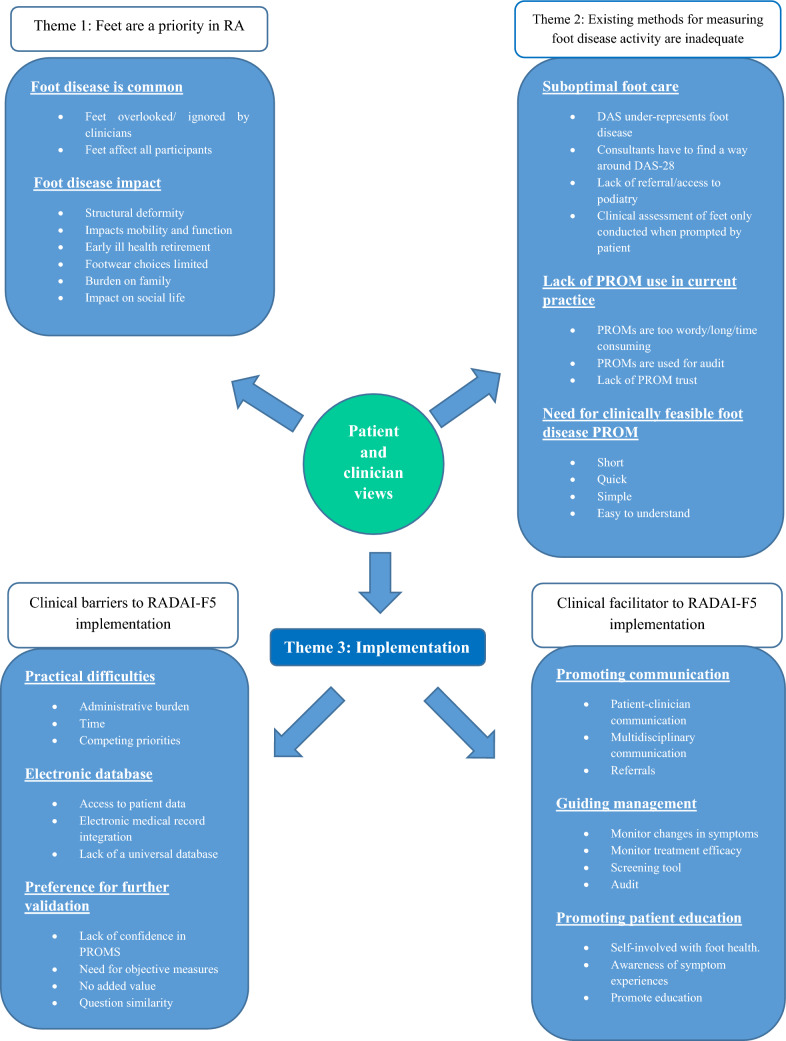
Overview of clinical utility of the RADAI-F5 for RA patients

The structured analysis resulted in three global themes; ‘Feet are a priority’, ‘Existing methods of measuring foot disease are inadequate’ and ‘implementation’. The global theme of ‘Implementation’ was drawn together to form 2 subordinate themes: ‘Facilitators to RADAI-F5 implementation’ and ‘Barriers to RADAI-F5 implementation’. Figure 1 illustrates the relationships between participant views and the final themes. These themes are explored further with illustrative participant quotes (Table 3) to support the findings. Contributing quotes from each RA participant and clinician to the overall themes are available within the article and its supplementary materials (2.1 and 2.2).

**Table 3:** Themes with respective quotes that emerged as part of the individual interviews to understand the clinical utility of the RADAI-F5

Theme	Quotations
Theme 1: Feet are important	“It's [Foot disease is] common and it's troublesome for patients …it's one of those things that people often will complain of when they first present and when the disease becomes more active again it's something that they will comment on not infrequently.” *(C16-Rheumatologist)*
	“I am very restricted, I can't walk very far, I use a mobility scooter.” *(P02-Patient)*
	“I went from being somebody that was quite dynamic and ran a business that employed 20 people, and I loved what I did …. I have had a massive drop in income and a massive drop in self-esteem.” *(P04-Patient)*
	“I couldn’t see my friends because I was always so tired and in pain. It is very lonely.” *(P07-Patient)*
Theme 2: Existing methods for measuring foot disease activity are inadequate	“The rheumatologist never really mentioned anything about my feet.” *(P01-Patient)*
	“The feet are under-represented in the clinical tools for assessing disease activity, and clinicians don't look at feet enough.” *(C11- Rheumatologist)*
	“Really frustrated that it (the feet) doesn't form part of the overall picture, and as I said, it's a systemic disease…. I've seen quite a lot of on the various chat lines that people are saying, you know, "my feet are bad, so why aren't they on the DAS," so it's not just me by any manner.” *(P03-Patient)*
	“I'm a bit frustrated… if you're getting people who are on the cusp of maybe being eligible for more advanced therapies, and you are then having to involve other members of staff … It is a minor barrier but we do get around it.” *(C16-Rheumatologist)*
	“We've tried numerous PROMS. Historically, I think probably it's time-consuming for our clinical consultations… and then writing up, the kind of administration side of things. I think we’re constructed by time”. *(C12-Podiatrist)*
	“I think it (the RADAI-F5) would really highlight the need for looking at feet because as soon as you've got an official test, but it puts on people radars” *(C17-Podiatrist)*
Theme 3a: RADAI-F5 facilitators	“If you give too many questions people get lost in amongst them all and maybe not able to be completed in the 10-min appointment. This(RADAI-F5) is nice and short.” *(P01-Patient)*
	“I think it (the RADAI-F5) will make that conversation easier for the advanced practitioner, but also make sure things aren't missed from a patient perspective. I think it improves the clinician-patient relationship” *(C15-Physiotherapist)*
	“I think it (using the RADAI-F5) could try and measure the success of the treatments that we are implicating.” *(C12-Podiatrist)*
	“It was only really when things start to get bad for my feet that I understood the importance of the feet.” *(P04-RA Patient)*
	“It would have been helpful to have this tool (RADAI-F5) so that I could have been more self-involved with my management and been aware of the long-term issues that occurred with my feet as a result of my RA” *(P07-RA Patient)*
Theme 3b: RADAI-F5 barriers	“I don't have a waiting area and I don't have anybody to hand a copy out… It would be difficult because like I said I don't have any admin.” *(C13-Podiatrist)*
	“I think it's time that is probably the big one that staff will probably try and push back on.” *(C12- Podiatrist)*
	“I mean, this (RADAI-F5) will obviously go along with other tools. You know, the blood tests and things as well.” *(P01-RA Patient)*
	“I think it could be useful as a patient tool, but the kind of integration into electronic patient records might be a stumbling block. *(C15-Physiotherapist).”*
	“I think there are things about coming to clinics that people change the nature of the problem …they just ramp up all the figures, everything is much worse.… I mean, sometimes a clinic is not really useful time to get PROMS. It is an artificial event…and it's much more useful to have these and accumulate some information between clinic times.” *(C11-Rheumatologist)*
	“Completing the RADAI-F5 on my mobile will be a constant reminder about how poorly I feel, and I don't want to do that.” *(P07-RA Patient)*
	“I suppose it's just about accessibility… we're going to cover base with people that are not so tech-friendly or have tech poverty.” *(C12-Podiatrist)*

### Feet are a priority in RA

All RA patients reported current or previous foot issues, and many RA participants recall their first RA symptoms being in their feet (C16). Foot discomfort, stiffness, oedema, numbness, joint deformity, and skin lesions such as corns and calluses were common complaints among RA participants. All RA participants also discussed the capacity to walk, with the majority stating that the disease has restricted their ability to walk a long distance (P02). As a result of RA-related foot disease, numerous patient participants had been forced to retire early or limit their work hours, impacting their financial and emotional health (P04). Foot difficulties also had a social impact on individuals, resulting in isolation and severe emotional and mental health costs for some (P07).

### Existing methods of measuring foot disease are inadequate

Although clinicians acknowledged that foot complaints were common and bothersome among this patient cohort, RA participants stated that foot-related conversations were infrequent during routine clinical visits (P01). They also reported that discussions around hands were prioritised over feet. Other patient participants were frustrated that the DAS-28 excluded the foot and ankle joints, which was seen as a considerable issue (P03), as it may lead to misclassification of disease activity. Rheumatologists attributed the absence of frequent foot exams to lack of accessibility to the feet, lack of foot assessment training, and, most crucially, lack of time during consultations. Nonetheless, clinicians expressed that it was difficult to alter medical treatment without a qualifying DAS-28 score, which caused frustration and additional workload for rheumatologists when foot symptoms were identified (C16).

Both clinicians and patients acknowledged that the lack of a clinically feasible method of measuring foot disease made referring RA patients in need of urgent consultations challenging (C11). Despite national recommendations for routine PROM usage in clinical practice, almost all practitioners reported not using PROMs regularly. This was mainly attributed to time constraints, administrative costs (C12), and rheumatologists cited competing objectives as a deterrent to PROM use. Nevertheless, some clinicians reported using PROMs for auditing rather than monitoring patient progress or communicating with patients about the impact of disease and therapy on their health. In this context, clinicians and patients stressed the necessity for a PROM like the RADAI-F5 to detect foot disease in RA patients (C17).

### RADAI-F5 facilitators

Participants agreed that this novel clinical PROM was easy to use and efficiently collected clinically relevant data for busy clinics (P01). Several participants also noted that RADAI-F5 implementation could improve therapeutic interactions between patients and clinicians by facilitating dialogues about foot health (C15), especially in the early RA patient cohort. All participants agreed that the RADAI-F5 could help to guide management and help screen patients for RA-related foot issues (C17). This could close a clinical care gap by demonstrating the critical role of the foot in the RA population, which is not captured by traditional disease activity measures such as the DAS-28. This may aid clinicians in screening RA feet, expediting patient visits, and tracking therapy efficacy over time (C12). Additionally, patients noted that seeing improvements in their RADAI-F5 scores may make them more receptive to treatment regimens. Other patients expressed difficulties with self-care due to a lack of knowledge regarding the impact of RA on the feet and the relationship between RA and foot health (P04). These patients felt that the RADAI-F5 had a beneficial effect on patient education and self-awareness regarding their feet. Other patients believed that by utilising the RADAI-F5, they would become more aware of their symptoms and thus be more proactive in self-management (P07).

### RADAI-F5 barriers

Numerous respondents raised reservations about the RADAI-F5's adoption. Concerns included the tool's administration, as AHPs may be unable to print, disseminate, and collect RADAI-F5 data without administrative support (C13). Additionally, clinicians will need to analyse, evaluate, and act on PROM data (C12), which will add time to their clinical appointments. Due to time restrictions, patient participants supported the completion of the RADAI-F5 in the waiting room prior to clinic sessions, believing that doing so would be more efficient and speedy. Nonetheless, clinicians preferred if patients completed the RADAI-F5 questionnaire at home, away from the added stress of the clinical environment. Additionally, it eliminated the possibility of patients falsifying their scores in order to influence the clinical appointment's outcome (C11).

Rheumatologists feared that the RADAI-F5 would be unable to detect comorbidities or changes in a patient's underlying illness, meaning that the tool could be reflecting indications and symptoms of a separate health issue. As a result, clinicians and patients recognised the importance of using objective measurements such as ultrasound imaging, clinical examinations, and inflammatory blood markers to further validate the RADAI-F5 tool's ability to detect rheumatoid arthritis-specific traits (P01). AHPs also emphasised the importance of recognising clinically significant score changes and action thresholds for specific patients. Rheumatologists suggested that clinically significant score shifts and action thresholds would help guide further management.

Rheumatologists, GPs, and AHPs, may not have access to the same electronic health record, posing potential access and reporting hurdles for RADAI-F5 data (C15). By associating personal choices with RADAI-F5 outcomes, clinicians hoped that integrating a mobile application (app) or ePROMs into the rheumatology service would increase patient involvement in their health and hold individuals accountable for their own foot health. Additionally, an app would minimise the time and administrative burden associated with RADAI-F5 adoption, increasing clinical efficiency. On the other hand, patients stressed that apps should not be used in place of in-person consultations. Additionally, several participants noted that using an app to record symptoms may exacerbate negative disease behaviours by continually reminding patients of the severity of their disease, which may have a detrimental influence on their mental health (P07). Access to digital technology and the internet was also mentioned as a barrier to mobile app uptake, especially among the elderly (C12).

## Discussion

As we strive to make foot healthcare delivery more patient‐centred and continue to encourage PROM collection as part of value-based initiatives in rheumatology, understanding how the RADAI-F5 may meet the needs of RA patients with foot disease is critical to establishing optimal foot health in this patient group. This study is the first, to our knowledge, to explore the clinical facilitators and barriers to new foot PROM implementation from RA patients, rheumatologists and AHPs perspectives. By identifying the perceived challenges and potential facilitators of using the RADAI-F5, our findings can help inform the development of effective strategies for RADAI-F5 implementation in RA as suggested in Table 4. The outcomes of this study revealed that rheumatologists frequently underestimate foot disease and that there may be unmet needs for better foot care [24]. Many RA patients believed their visits were driven by the DAS-28, which is concerning considering people in DAS-28 remission can have active synovitis in their foot joints [25, 26]. A shortage of time during consultations, accessibility to feet, and difficulties measuring foot joints even with training, were all noted as competing domains. This is corroborated by prior research [27, 28] and implies rheumatologists may require more training on examination of foot and ankle.

**Table 4:** Effective RADAI-F5 implementation strategies

Perceived barriers	Effective RADAI-F5 implementation strategies
Lack of electronic databases	Integration of PROMs data into health record App
Practical implementation difficulties	EPROMs Mobile App Administration of PROMs in waiting area
Lack of PROM	Education on PROM purpose Association with ultrasound Association with clinical examination

Although the 66 joint counts have been used previously to assess patients with RA since it includes the foot and ankles [29], performing 66 joint counts is time demanding and application in routine clinical settings is thus limited [30]. All clinicians acknowledged a need for a widely used, validated and clinically feasible method for the early detection and assessment of foot disease activity in RA. Therefore, an RA-specific foot PROM, such as the RADAI-F5, could be an effective alternative to highlight and screen feet in the current patient population. The RADAI-F5's short length, format, and simplicity of language is seen as a desirable PROM property as these qualities produce higher response and completion rates [31, 32].

Systematic reviews of PROM use in health care settings discovered that PROMs improve clinical diagnosis through positively impacting patient-physician communication [33, 34]. This was supported by the majority of participants (*n* = 13), agreeing that employing the RADAI-F5 may facilitate a more holistic, patient-centred approach to care by improving patient-clinician dialogue and recognising aspects of foot health that is important to the patient. Using PROMS to improve patient–clinician trust has been established in numerous studies [35, 36], and these strong relationships may result in better patient outcomes and promote 'humanising care' [37].

Using PROMs like the RADAI-F5 could educate RA patients and promote self-management of foot health. This increased knowledge, along with constant feedback from monitoring their RADAI-F5 score, may improve perceived foot health control. Ndosi et al. [38] found that PROMs can deliver an educational programme tailored to a patient's needs and identify potential goals for promoting patient autonomy while facilitating shared decision-making about treatment plans between patients and clinicians, increasing the likelihood that patients will adhere to the chosen treatment plan [39]. The RADAI-F5 data can also identify poor foot disease, allowing clinicians to discuss when treatment is not progressing as intended. The RADAI-F5 scores can then be used to track the impact of changes on treatment progress. This may save time ordering additional testing and referring patients [40, 41], providing more efficient care pathways.

Despite rheumatologists' recognition of the RADAI-F5's value in promoting patient-centred care, various barriers to the tool's clinical integration were identified, including administrative burden and time-limited appointments. Although completion of PROMs in waiting areas before clinical consultations has been supported by our RA participants and recommended in some research [42], this may not be recommended due to the risk of bias since individuals attending more visits may have worse outcomes, or maybe worsening their PROM scores to elicit clinician response [43]. Adopting the RADAI-F5 in clinical settings may be hampered by logistical and technological issues. Participants recommended apps and ePROMs to make RADAI-F5 data more accessible while reducing clinician and administrative workload [44]. Although the clinical use of apps and ePROMs is still in its infancy, research demonstrates that the general population can use systems with little difficulty [45, 46]. Moreover, collecting ePROMs and remotely sharing symptom data with healthcare providers may be important during pandemics like COVID-19. It is necessary to secure the administrative and financial support to implement the technology needed for ePROM assessment and real‐time scoring. Routine PROM data collection will be successful only if a system is created that limits the added load on clinicians [47] and requires deliberate institutional prioritisation from hospital trusts. While our study's patients endorsed the utilisation of ePROMs, many highlighted that virtual clinics should not replace regular face-to-face consultations. Primdahl et al. 2020 [19] supported this notion by stating that dialogue with patients is critical to complement PROM data in routine care.

Without comparable objective data, the RADAI-F5 may be enlightening for clinicians but may not be sufficient to adjust therapy based on individual patient results. Additionally, rheumatologists indicated that altering medication based on the RADAI-F5 was deemed risky and costly unless additional objective metrics validated the PROM. However, it should be emphasised that early diagnosis of foot disease and appropriate treatment may be helpful in reducing the need for use of expensive treatments. Nonetheless, to address these issues, clinicians and rheumatologists would prefer to see additional validation work using objective measures such as erythrocyte sedimentation rate (ESR) and C-reactive protein (CRP) biomarkers or to examine the relationship between the RADAI-F5 and ultrasound imaging and clinical examination, which will be explored in future studies.

Our study's strengths include its systematic approach and comprehensive review of the barriers and facilitators of new foot PROM implementation from the perspectives of patients, rheumatologists, and AHPs. The topic guide also incorporated information from RA patients and clinicians with varying backgrounds and expertise, resulting in a diverse spectrum of topics and questions. This study used theme saturation and investigator triangulation to improve the findings' credibility and dependability while reducing bias [48]. Several limitations to our study are also worth considering. There a risk of selection bias as individuals who opted to participate in the study may have had a higher level of foot disease and had negative experiences with rheumatology departments. Some participants were also enlisted through the study team's professional networks and social media advertisements, leading to self-selection bias. Additionally, because some AHP participants were aware of the RADAI-F5 project's long-term objectives, our findings could be skewed by respondent bias [49]. This study only included members of the online community, which may restrict the generalizability of the study's findings. Another potential drawback is the inclusion of a greater proportion of female participants than male individuals. This, however, may be a reflection of the gender disparity in the prevalence of rheumatoid arthritis, which is more common in women. Additionally, because all participants were British, our findings may not be transferable to other countries.

Our study highlights the RADAI-F5 as a potentially significant clinical tool for RA patients and clinicians and highlights different implementation strategies. Despite the tools' limitations, clinicians were enthusiastic about their ability to improve care and promote a "treat-to-target" strategy for RA of the foot. Our findings show that the RADAI-F5 must be clinically validated and integrated before being widely used in rheumatic clinics. These innovative findings emphasise the critical relevance of involving clinicians and patients in future successful implementation of PROMs.

## Supplementary Information

Below is the link to the electronic supplementary material.

Supplementary file1 (DOCX 37 KB)

## Data Availability

The authors confirm that the data supporting the findings of this study are available within the article and its supplementary materials.
