# Supplementary material for: Patients’ and clinicians’ perspectives on the clinical utility of the Rheumatoid Arthritis Foot Disease Activity Index
Source: Rheumatol Int. 2022 May 27;42(10):1807–17. doi: 10.1007/s00296-022-05147-8 (PMC9136191; doi:10.1007/s00296-022-05147-8)
Supplement: Supplementary file 1 — Supplementary file1 (DOCX 37 KB) [file 296_2022_5147_MOESM1_ESM.docx]

# Appendix 1.1: Interview topic guide (patients)

**General experience of arthritis and foot disease**

1. “Can you tell me a bit about how your arthritis affects you?”
2. “Could you tell me about any changes to your feet since developing RA??”
3. “Can you explain how your foot problems affects you and your day to day life?”
4. How have your feet and any problems affected your quality of life? Prompts (your daily activity? Activity levels?

**Patient perception of foot disease:**

1. **“**Does your doctor discuss your DAS score with you and do you know what it means?”
2. “Can you give me an example of a time that your doctor or rheumatologist has discussed your DAS-score with you?”
3. “How often does your rheumatologist or doctor discuss your DAS score with you?”

**Foot care assessment and access:**

1. “How do you decide when it is time to seek help for your foot problems?”
2. “How comfortable are you in discussing your foot problems with your healthcare team?”
   1. Why are you comfortable/ not comfortable in discussing your foot problems?”
   2. “Do you always get to talk about your feet when you need to? If not, why? If so, what happens next?”
3. “Has anyone examined your feet since developing RA?”
   1. “Who examines your feet?”
   2. “How regularly does someone examine your feet?”
   3. “Do you feel that your foot problems are being examined appropriately?”
4. “If your feet are bad and the doctor knows, what do they do for you?”

“Have you had any experience of seeing a podiatrist, physiotherapist or orthotist for treatment for your feet?”

“Why did you get treatment?”

“Have those treatments been beneficial? How have the treatments been beneficial?”

“Has your medication changed because your feet have been bad?”

**RADAI-F5 tool:**

1) “What did you like about the tool? What did you not like?”

1. Are there any questions in the tool that are unclear or confusing?
2. “What are your thoughts on completing the questionnaire in the waiting area before seeing a doctor or rheumatologist?”
   1. How would you feel about your questionnaire being handed to the doctor/ rheumatologist to be discussed during your appointment?
3. “Would you want to know your score and what it means?”
   1. “would you write it down somewhere?”
   2. “would you consider using it to monitor your feet?”
   3. “How would you feel about your RADAI-F5 scores being used by other members of your healthcare team?”
4. “How do you think this tool would help in a telephone/video consultation during the Covid-19 pandemic?”

**Diagram:**

1. “How would you feel about indicating which joints are tender or swollen on a diagram of the feet?”

**App:**

1. “How would you feel about using an app on your smartphone/ laptop/ tablet to self-monitor your RADAI score?”
2. “Are there any reasons why you would not want to use an app on a smartphone/ laptop/ tablet?” (privacy, intrusion, difficulty with using hands etc.)?

**Closing question:**

**1) “**Is there anything else that you feel is important that we haven’t discussed?”

# Appendix 1.2: Interview topic guide (clinicians)

**Foot disease in RA and foot assessment:**

1. What is your understanding of foot disease activity in rheumatoid arthritis?
2. How do you believe that foot disease is represented by current measures of disease activity?”
3. How does it make you feel that feet are not included in the DAS-28?
4. “How frequently do patients discuss their foot health needs with you?
   1. “Do you believe patients have sufficient opportunity to discuss their foot health needs during appointments? Why?”
5. “What are your reasons for choosing whether to examine feet?”
   1. “How comfortable are you in assessing feet? Do you feel clinically competent in assessing feet?”
   2. “How frequently do you examine feet of RA patients?”
   3. “How much of your decision to not assess the feet are driven by the DAS-28?”
   4. “Is this driven by what the patients are reporting?”

**PROMs:**

1. “For which reasons do you currently use RA-specific Patient reported outcome measures (PROMS) in routine clinics?” –

*Prompts:* *screening, monitoring, shared decision making etc.*

- 1. “How do you use these?”
  2. “How frequently do you use PROMs?”
  3. “What influences your choice on when to use PROMs?”- RA. internal comparative- monitor disease activity- confounders. (inadequate to measure)

1. “How do you use the data gained from the PROMs to inform care or guide management of patients?”
   1. “Are there any barriers to this?”
   2. “Where/How do you record any patient-reported outcomes?” (*Trakcare, Portal)*

**RADAI-F5 Tool:**

1. “Would this tool be useful in your practice?”
2. “If yes/no, Why?”
3. “How would you use the tool in your practice?” (assessment of feet, referring, screening etc.)
4. “What are the advantages of implementing the RADAI-F5 in your routine clinical practice?”.
5. “What are the barriers of implementing the RADAI-F5 in your routine clinical practice?”
6. “Do you think the tool is clinically feasible?”
7. “How do you think the RADAI-F5 could be used to inform or guide subsequent further assessment or management including referrals?”
8. Patient-reported outcomes have become increasingly important during the covid-19 pandemic where there is an increase in telephone and video consultations. With this in mind:

“How may the RADAI-F5 be incorporated into telephone/video appointments?”

**Diagram:**

Due to Covid-19, many appointments have been moved to telephone or video consultations. It can sometimes be difficult for patients to explain where they are experiencing symptoms using just words. Keeping this in mind:

1. “How would you feel about the inclusion of a diagram to highlight which joints or areas of the foot were tender/swollen?”

1. “How would this diagrammatic information aid your practice?”
2. In your experience, how accurate do you think patients are at locating pain?
3. How do you believe patients can differentiate between RA and secondary comorbidity pain?

**App:**

1. “How would you feel about patients using an app via a smartphone/laptop/table to self-monitor their RADAI score?”

1. “Are there any reasons why you would not want participants to use an app on a smartphone/tablet/laptop?”

**Closing question:**

**1) “**Is there anything else that you feel is important that we haven’t mentioned or discussed?”

# Appendix 2.2- Contributing quotes from each RA participant to overall themes

|  | **P01** | **P02** | **P03** | **P04** | **P05** | **P06** | **P07** | **P08** |
| --- | --- | --- | --- | --- | --- | --- | --- | --- |
| **Theme 1:** Feet are a priority | I had to take early retirement due to ill health, so I have taken partial ill health retirement caused by my arthritis…  Obviously like most people with arthritis, I often get corns or you know you get slightly deformed toes, with one foot being worse than the other…  That’s been one of the hardest things (with her RA), replacing shoes and finding shoes that fit when you can't try them on…. | My left ankle is fused; my right ankle is beginning to fuse… I now notice that I walk different…you wouldn't have known that I have rheumatoid arthritis, whereas now you can tell. I am very restricted, I can't walk very far, I use a mobility scooter…  I use the wheelchair in the house because, in the mornings, I cannot walk at all. I can't even stand in the mornings because I am in that much pain…  I gave up work after I had my daughter and medically retired because my body has got so much damage now… | I had retired at the time of having RA…  I have difficulty walking the distances that I could do previously, and I have fatigue…  My feet have suffered from overtime. In those nine years, I have fallen arches. When I'm walking, there is considerable pain that's associated with the development of RA…  In terms of distance, I used to be a runner. I don't run now... | It's (RA) life-changing. It's just limited my activity so much…  The biggest change for me was that I did have a business that I ran for over 20 years, and because I was so poorly and couldn't cope with any kind of stress as it flares everything…  My feet were so sore that I couldn't walk to the end of the street and had to turn back because my feet were so painful…  I feel that the things that I would say like “Let us go for a walk, and I feel better", some days I can't do that, so I don't feel better. Therefore, that reduces my social interactions, so let's say that I can't meet a friend for a walk or I just can't go into nature, which would be something that I would have done in the past. | I am very limited and very restrictive movement…  Initially, when my rheumatoid started, it started apparently in my right ankle, and I still have problems with that. I feel my ankles are solid, I can get a wee bit to move it up and down, but my feet……yeah, I can't stand now at all… | I think prior to changing into the job that I have now, which is much more of a kind of light touch consultancy job, I was very stressed and really suffering quite badly physically from the disease…  You tend to get it (swelling) on the underside of the foot, typically under the big toe, basically in that joint that connects it to the body of the foot, it feels like it is crunching and stiff after walking a lot…  To be honest standing for long periods of time can actually be really not good for me… | I would say that I tried to take my own life at the turn of the year because of the pain. I was in that much pain…  The pain in my feet can be so acute that it is not unusual for me to sit in really bad winter months have my heating up at 27 degrees with socks on, and I spend days on the sofa. That is just not the person that I was but the first person I'm increasingly becoming…  I am retired. I retired five years ago. I loved my job, my job loved me, but I just became a very unreliable employee because of my foot pain… | [My RA] has affected it[feet] hugely.  I work in school, so I was working full time and I couldn't cope working. Then I reduced my hours to three days. I do get tired a lot…  It hugely impacts how I am and I think I'm still getting used to that because I still remember how I was and how I used to enjoy life. So it's now enjoying life in a very different way. It's hard, but it's getting easier as I get used to it more…  My toes. I struggle a lot with my toes. I don't get a huge amount of swelling but I do get a lot of tenderness and stiffness. I get a lot of pain going across the top of the foot and in my toes. My toes constantly just go into spasm and they just stick up and I can't do anything with them. Sometimes when walking, I will suddenly just won't be able to walk because it's stuck and really painful for it to actually move... |
| **Theme 2:** Existing methods for measuring foot disease are inadequate | Very confused (that feet are not included in DAS-28), because I am affected by my feet but I seem to forget to mention it…  The rheumatologist never really mentioned anything about my feet. But I think I am very knowledgeable about it now and like I said, he might only have 10 minutes so maybe cannot provide that much information…  They always check my hands or you know apply pressure in your hands and check your joints and those (*in hands)*, but I can't say they ever really look at your feet to be perfectly honest…. | No, but she does get you to take off your socks and shoes, and she will look at your ankle placement. I would say that the most time is actually spent on your upper body and your bigger joints…  Considering the amount of damage that I have, it (the DAS-28) should definitely include [the feet]*.* I can cope with my elbow problems, and my fingers are swollen, but the feet really restrict you because it restricts my life so much… | There is a commonly used tool, which you will know about called the DAS, which doesn't have feet in it. It's really very poor, but there is no measurement of feet…  If you don't have your feet measured and they are terrible, yeah, you're not going to get the benefit of the more effective drugs and biologics… So I think it's an excellent idea to push for feet to be included… | I don't understand. It's a whole-body disease, so I don't understand why they wouldn't include the feet. I think it's really unfair that it's not included in the DAS score…  I mean, my toes were separating, like the disease was active because you can see my toes were swollen and spreading*.* But he said, "it is not hot, and your markers are coming back normal, so it's not active". I have to admit I haven't had a great experience lately…  Well, I think it makes you feel less likely to want to tell them [about your foot problems] because you feel really belittled. You're not taken seriously by the rheumatologist, you feel like a fraud and that you are going crazy in your head. You know, you come back home, and you doubt yourself and think that you are a hypochondriac making things up. You know it's horrible… | I would have thought that it (the feet) should have been included (in the DAS-28) or at least in another form like the one you've got at the moment. For me personally, it is my feet and ankles that….if you see ankles as solid like mine, it does affect the way you walk, obviously… | I would imagine the two main areas that people are affected with the most are feet and hands. I'm kind of really surprised to have to say that it isn't included (in the DAS-28), so it probably should be a figure or in their measurement...  They're (rheumatologists) not particularly proactive with the feet. So no, I suspect unless I said "I've got a gammy foot", then nobody would start looking at them… | Why doesn't the DAS-28 include the ankle, or why do I not know that it doesn't. Is there something else that I should be having done because my ankles and my feet are very swollen all of the time? Is there another test I should be getting, and why am I not getting that? | My first consultant who I asked to move away from, said that it couldn't possibly be RA because it wasn't part of the joints that are supposed to suffer from RA, but that is where it affects my feet…   I do feel that the feet are very sort of underrated with the RA. It is all about "Oh, I want help with my hands", and I can see the OT. If I need help for another part of my body, I can see my physio but with the feet....No one seems to push how important the feet are, so I do believe that the feet should be included in the DAS-28… |
| **Subordinate theme 3:** Clinical facilitators to RADAI-F5 | This tool can be used to explain where your foot pain is to help them make a better informed decision. I think there are a lot of people out there who haven't been able to. If they have had a lot of foot pain, they will not have had a chance to speak to anybody about it….  I think it's (the RADAI-F5) quite good to make you more aware of your feet, because I think it's like all these things you're inclined to. You don't ignore it; you learn to live with it. When you sent the form, I started to look back at my feet and I am aware that when my arthritis isn't right, my feet are wrong….  If you give too many questions people get lost in amongst them all and maybe not able to be completed in the 10-minute appointment. This is nice and short…. | If you are given this tool when you have an illness, it is always in your brain that you need to look after that part of your body and talk about it to my rheumatologist….  I wish I would have known about my feet before so I could have pushed for more help…I went from 0-100; somebody like me should have been caught quicker and been told to get into these feet before they end up with all these deformities. That never happened to me. It wasn't just treated like the rest of my body. It would have been helpful to have this tool so that I could have been more self-involved with my management….  This one was nice and short. It also takes no time to fill this in; it takes literally a few seconds. I don't even think the kettle had boiled by the time I completed it *(laughs)*. So, it was very quick so should not take up much of the clinic time….. | You can see if it is (The RADAI-F5 scores) in the same place or if it is increasing or decreasing. I could then reflect on what I did around that the affected things….  Yeah, the five questions were pretty easy to understand. And you had it “thinking only of your feet”, which I think is necessary because people will perhaps use it for other things….  Sadly, my experience in Scotland is that there are too few rheumatologists to keep on track with the changes that are affecting the patients. It (The RADAI-F5) would give a consistent basis for the rheumatologist, podiatrist and orthotists to compare how you were before with how you are now. …. | It would make me feel more in control if I was doing the self-assessment via a tool like this. Then I could go in and say, “These are the things that I want you to focus on now….  You can see if it is (The RADAI-F5 scores) in the same place or if it is increasing or decreasing. I could then reflect on what I did around that the affected things….. | I would be happy to discuss whatever I put on the form with them (rheumatologists) as a starting point. If my feet had been sore in the last few days, I would probably want to discuss that using the form (RADAI-F5) ….  This could help the consultants focus on the feet if it was the more symptomatic part of the body. Consultants do not have a lot of time to go through every part of the body, so using this could help focus on the feet and help set a plan or treatment focused on the foot….  Well, it was easy to fill in. The questions are clear. You know, sometimes when you get these forms, you think, “what does that mean?”. But I understood all the questions in the form that you provided…. | I think that it potentially prompts joint-specific discussions. It's almost like a tool to perhaps kind of just help the discussion be a bit more efficacious ……  If you've got a question here with 500 questions on it, nobody fills it in. If you've got a questionnaire with five questions, everyone will at least have a go….. | I would hope that we would go through the tool and take a question at a time and talk it through thoroughly while an examination was being done….  Because the RADAI-F5 asks about the joint tenderness and the pain and foot pain and my foot health, so I think it will help me focus on my feet and encourage more treatments that I could do myself….  It was succinct, it was very easily understood, it was just very black and white and short and to the point…. | There has to be open communication between the whole health team…..  It (RADAI-F5) was definitely a good tool to have so you can monitor your feet, so you know whatever time you had your medication sort of. Well, it's three months really before it starts to kick in, isn't it? It could be a useful tool to then look back and think, "Actually, that was actually really mild compared to what I am now….  I thought it was really easy, really simple. You know it does exactly what it says. The things on there (*questions)* it's straightforward. It's self-explanatory. There's nothing on there that I think would confuse anybody. It's not long, it's everything you need, and it is there and quick in clinics….. |
| **Subordinate theme 4:** Clinical barriers to RADAI-F5 | He only gets to see you for 10 minutes. It's not very long, you know?  I mean, this will obviously go along with other tools. You know, the blood tests and things as well. | You don't actually get a great deal of time to talk (to your rheumatologist), although she does try her best to accommodate you most of the time you are referred to a surgeon, a physio or the podiatrist. | It might make it more acceptable to the clinicians for there to be an independent, evidence-based measure included in the RADAI-F5. That struck me as being perhaps, or something which would present you with a difficulty in persuading rheumatologists that this is not just measured by the patient, but also as an independent source of information |  | They have got very limited time during an appointment; they might not always have the time to go over every single one of the question | I mean, it's just building up another pool of details on that and your response to a tool rather than the rheumatologists. Is it significantly less accurate? You're still going to have all the DMARD and blood monitoring thing so you can have all the inflammation markers and everything, so they'll have some data. | If information isn't picked up by the nurses in Rheumatology Day Ward, it is picked up by the secretary, but they are very busy. You just wonder if they have the time to hand you the form and collect it again. | There was some question similarity between question 2 and 3 and there was not a lot on walking and effects on daily activity. |

# Appendix 2.2- Contributing quotes from each clinician participant to overall themes

|  | **C10** | **C11** | **C12** | **C13** | **C14** | **C15** | **C16** | **C17** |
| --- | --- | --- | --- | --- | --- | --- | --- | --- |
| **Theme 1:** Feet are a priority | If they have other joints that are more painful at the moment in time on top of their feet, that one will take preference to another, and the feet will be overlooked… | It's (the feet) a frequent issue at consultations.  It obviously depends on the extent of that person's disease, but certainly you could imagine that the feet would be mentioned in up to 50% of our consultations… | My understanding is the impact of feet that it has on a patients’ disability. It creates a disability that comes to their foot function everyday sort of like activities that it has a major impact on. I suppose functionally but also to take into account sort of the biopsychosocial model, it just impacts on their life as a whole…  I have had lots of experience where the DAS-28 looks positive but the patient sitting in front of me hasn't given me that same level of feedback in relation to their feet… | N/A | I don't really see very much with the early in inflammatory arthritis, so the new patients and the flares and the synovitis and that part of rheumatology. I mainly see the referrals from rheumatology for those patients that might be new or have been with the rheumatology consultants for a while and any foot problems that come in are kind of sent my way. So primarily, it's the mechanical problems with the foot that I tend to see rather than the inflammatory issues… | I know that the feet are undertreated and probably under assessed area nationally for people with RA. Also, my understanding is there's a lot of potential overlap of the erosive disease causing secondary degenerative disease, so even in a theoretically well-controlled rheumatoid patient, there could well still be foot problems there. I suppose, as the physiotherapist, if there are problems with the feet that impacts on everything else, like our patients’ ability to do with regards to cardiovascular fitness and that kind of thing. So it's a crucial part, I think, of the overall package of care and management. | It's common and it's troublesome for patients because it can have a major impact on their mobility. Pain control can be an issue…  It's particularly difficult with the DAS-28 because you do get some people who predominantly have foot and ankle disease and that won't be represented really at all in their DAS score…  I would spend much more time on the upper limb that I would on the lower limb with students as well, never mind trainees… | N/A |
| **Theme 2:** Existing methods for measuring foot disease are inadequate | I think in the DAS-28, it (the feet) is measured very poorly, actually. We often find that the disease looks as though it is in remission according to the DAS, but they have still got problems with their feet. So current measures in terms of just clinical measures as in the DAS-28 is just not reflective at all of any problems within the feet…  I mean, we don't use PROMS as much in South Manchester.  It makes me feel that we're not providing the best service to the patient because we are completely omitting anything below the knee joint. I don't think it's reflective of the person as a whole; it's just a snapshot of what they are actually seeing at that time without including the feet… | Well, this is under-represented in the clinical tools for assessing disease activity, and that clinicians don't look at feet enough…  People often have substantial foot disease and not so much in their hands, so it creates problems which we find ways around. But it's just irritating and frustrating for everybody…  There is sufficient time to discuss whatever their priority is. So if they mention that *(feet)* as their priority, we will talk about that. You know, I think if it's not a big issue, then they will not mention it… | We don't routinely use any PROMS other than the traditional VAS scores.  We do count joints and document joint involvement, but it's more of a kind of a written X bar detailing clinical history rather than using any structured PROMS…  We've tried numerous PROMS. Historically, I think probably it's time-consuming for our clinical consultations. It is hard to try and capture all of the aspects of history taking, assessment, treatment and then writing up, the kind of administration side of things.  I think were constructed by time, I suppose…. | Foot disease is currently not measured or represented by current measures of disease activity  because it doesn't really. There’s nothing that's specific to focus just on the feet… | You've already said about the DAS-28, which doesn't involve the feet at all, so there isn’t any real sort of outcome measures that are used. In Salford, we use the Minemop Outcome measure which you measure yourself and is a medical outcome profile. It's a kind of a broad generalized outcome profile. It doesn't particularly pinpoint the feet as such. Yeah, I do think there's something missing (in terms of patient-reported outcomes). We don't use any of the long-winded foot outcome profiles. We just don't have the time to use anything like that really… | I think it's a real missed opportunity, and I think we do the screening tools we are told to do and probably not much more. A well-informed MDT kind of positive clinician will consider foot problems every time they see a patient but perhaps those that are less experienced or maybe the nurse-led clinic will just do the DAS-28 because that's what they've been asked to do and foot problems don't come into that, so it's a missed opportunity to make sure we screen for that…  We use the HAQ. We have tried the MSK-HQ but we didn't progress with that. We have tried EQ-5D, again we didn't particularly progress with that. For RA.... Pain VAS as a part of the DAS-28 is probably the two other embarrassingly... | I think within practice it's something that we're all quite aware of, so we'll regularly ask patients about their feet but for scoring and eligibility, it does give a little bit of difficulty…  If you're getting people who are on the cusp of maybe being eligible for more advanced therapies, and you are then having to make an estimation of how bad their foot disease. As I said, involving other members of staff or other members of staff may have to involve you because again they got the same problem. It is a minor barrier but we do get around it… | Foot disease is  not represented. It is basically ankle up, and it's like the feet don't matter…  I think it's (feet) just not at the forefront of their mind because of that (exclusion from the DAS-28), and because it's not on that checklist…  I don't think my two that do MSK do (use PROMs) currently. I don't think they have separate ones for RA… |
| **Subordinate theme 3:** Clinical facilitators to RADAI-F5 | I just like the ease of being able to fill it (The RADAI-F5) out, to be honest. It's just simple. They are all extremely quick questions, and it gives you a very quick overview of what's been happening…  his (The RADAI-F5) should form part of your patient education because you are building a relationship with the patient where you are acknowledging their foot problems. You know...we will be offering treatment in response to that, but we are also offering advice and building that patient relationship…  I think I would use it for new patients but also for existing patients just to monitor progress from one appointment to the next. I think with the new early RA patients; I think we would use this tool to just monitor them to see how they were going in terms of the global disease as well rather than rushing things… | Well, it's (the RADAI-F5) simple. It is not complex in both the number question and the terminology is easy for folks and it's nice that it is a Likert scale or something similar to the Likert scale. Yep, it looks good as a PROM that could be quick in clinics…  You may have to have it as some type of internal comparative for the patient's own baseline and see whether it was going up or down… | I thought it was really simplistic, easy to use, and easy to calculate…  I think it could also encourage patient communication with clinicians and make certain that we are facilitating outcome driven care…  I think it would be to try and measure the success of the treatments that we are implicating… | I think that's great and I love the fact it's very short. We've got sort of half an hour to 40 minutes to do everything else, so I can't spend 10 minutes on this. That's how I really appreciate that it is concise. It's the fact it's five questions and it's dead easy to score…  it would be nice to (*use RADAI-F5 as a monitoring tool)* because it would be a quantifiable thing that you can actually sit and see improvement…  Patient-related outcome measures are really important to my work and help guide management… | That there are only five questions, and it's a simple scale of 0-10 and quite easy for them for me to understand…  It (the RADAI-F5) will assist in referral decisions with regards to AHP services, or like I said, clinical psychology or anything else. It will assist in disease medication, modifying and general management plan review… | It's short. It's fairly easy to fill out. I think 5 questions, scoring zero to 10, it's quite clear what the bounds are at the 0 and 10. I think you probably could ask 1000 questions and still not know everything you need to know, but only get down to five is much appreciated…  I think self-monitoring is very good for the patient…  Do I make sure I get shoes and socks off for all my patients? Probably not as much as I should do, but if they mention it subjectively, I'll assess it. Yeah, that (the RADAI-F5) could help with the conversation if we're looking at very specific treatment issues… | It was short*.* So it's only 5 questions. It's very straightforward. I think it's easy for patients to understand the scales and at least I don't need to get my ruler out to measure what the score is, which you do for other things. And yeah, those are the main things... Short and easy to score so quick to use in clinic…  I think that could be quite useful because that's often where the conversation starts…  I could see that (The RADAI-F5) being useful if you're doing an intervention, particularly aimed at the feet, then following it up with this (RADAI-F5).  You know if you've got a pre and a post and then what level of improvement you're looking for. I could see that being useful… | I thought it looked quite easy to use. It was easy for a patient to understand...the patient would understand the wording of it and things as well. I think it is clinically feasible. It's not going to take a long time to do. I think that'll be quite easy to implement into a clinic, and it is at a level of consultant could understand…  It is nice because it helped track your patterns and things as well.  It sort of made the patterns for you, which was really good because it helps encourage self-management...  Let's say if it was an MSK appointment, I would be using this (The RADAI-F5) every appointment to monitor the changes and the orthotics and the treatment that was being done…  I think it would really highlight the need for looking at feet because as soon as you've got an official test, but it puts on people radars… |
| **Subordinate theme 4:** Clinical barriers to RADAI-F5 | I think, to be honest, it is more the time restraints and what we would do with that information...  It is hard because we are not all on the same electronic system…  However, there may be some concerns that it needs to be compared to bloods or what we tend to do, which is ultrasound imaging of the joints. I think they would want to find out if this tool compares to labs first. I don't think it can be totally used on its own; I think it needs to be used as part of the clinical assessment as well… | I think there are some time issues with clinicians Clearly there would have to be some way in which clinicians would not be overburdened with various PROM scores, flagging up, and so on, it just causes more administrative work… | I think you suppose in terms of barriers, I think it's time that is probably the big one that staff will probably try and push back on…  Yeah they don't have access to Trakcare, which could be an issue when reporting RADAI-F5 results... | I don't have a waiting area and I don't have anybody to hand a copy out. I don't have any admin so I can't do that before they come to the room…    Bear in mind, the consultants and the nurses are on a different note system than I'm on, so the two wouldn't be able to work together anyway, so that's a problem…  I think it is important that you compare the RADAI-F5 so that we can look at disease ratios, we can look at bloods, we can look at X-Rays… | It would be so much easier for me rather than writing everything out and reduces that administrative side of things or should I say that barrier aspect. I think the consultants were possibly a little bit apprehensive and you could see a few barriers and thinking this is going to take time and I think they're really pushed for time...  It (An app) cuts out the middleman of trying to get through admin and that administrative burden… | So I suppose it's not necessarily a lack of desire to do them, but it's the realism of how much you can complete with a patient during a short consult and the administrative factor…  So I think it could be useful as a patient tool, but the kind of integration into electronic patient records might be a stumbling block…  We probably do bloods or imaging in the interim and just see if there is an overall disease activity score… | Time*.* Time is the biggy. It's finding enough time in clinic appointments that are stretched…  Now this (RADAI-F5) could obviously be done and scored before they came in to see me, but that means somebody has got to be doing this scoring and the interpreting of it… | They (podiatrists) don't see the point in that information being disclosed and the admin time to get it all entered. We have got a 6 practitioner clinic, so that is hundreds of patients every week that would then have to find the admin resources to put that on…  It is the patients’ perspective; it can be difficult to trust. We have to still do clinical examinations |
